# *Anaplasma phagocytophilum* APH0032 Is Exposed on the Cytosolic Face of the Pathogen-Occupied Vacuole and Co-opts Host Cell SUMOylation

**DOI:** 10.3389/fcimb.2016.00108

**Published:** 2016-09-22

**Authors:** Aminat T. Oki, Bernice Huang, Andrea R. Beyer, Levi J. May, Hilary K. Truchan, Naomi J. Walker, Nathan L. Galloway, Dori L. Borjesson, Jason A. Carlyon

**Affiliations:** ^1^Department of Microbiology and Immunology, Virginia Commonwealth University Medical Center, School of MedicineRichmond, VA, USA; ^2^Department of Pathology, Microbiology, and Immunology, University of California School of Veterinary MedicineDavis, CA, USA

**Keywords:** *Anaplasmataceae*, intracellular bacteria, SUMOylation, inclusion membrane protein, *Rickettsia*

## Abstract

*Anaplasma phagocytophilum*, a member of the family *Anaplasmataceae* and the obligate intracellular bacterium that causes granulocytic anaplasmosis, resides in a host cell-derived vacuole. Bacterial proteins that localize to the *A. phagocytophilum*-occupied vacuole membrane (AVM) are critical host-pathogen interfaces. Of the few bacterial AVM proteins that have been identified, the domains responsible for AVM localization and the host cell pathways that they co-opt are poorly defined. APH0032 is an effector that is expressed and localizes to the AVM late during the infection cycle. Herein, the APH0032 domain that is essential for associating with host cell membranes was mapped. Immunofluorescent labeling of infected cells that had been differentially permeabilized confirmed that APH0032 is exposed on the AVM's cytosolic face, signifying its potential to interface with host cell processes. SUMOylation is the covalent attachment of a member of the small ubiquitin-like modifier (SUMO) family of proteins to lysines in target substrates. Previous work from our laboratory determined that SUMOylation is important for *A. phagocytophilum* survival and that SUMOylated proteins decorate the AVM. Algorithmic prediction analyses identified APH0032 as a candidate for SUMOylation. Endogenous APH0032 was precipitated from infected cells using a SUMO affinity matrix, confirming that the effector co-opts SUMOylation during infection. APH0032 pronouncedly colocalized with SUMO1, but not SUMO2/3 moieties on the AVM. Ectopic expression of APH0032 in *A. phagocytophilum* infected host cells significantly boosted the bacterial load. This study delineates the first domain of any *Anaplasmataceae* protein that is essential for associating with the pathogen-occupied vacuole membrane, demonstrates the importance of APH0032 to infection, and identifies it as the second *A. phagocytophilum* effector that co-opts SUMOylation, thus underscoring the relevance of this post-translational modification to infection.

## Introduction

Bacterial proteins that decorate the cytosolic faces of pathogen-occupied vacuole membranes are critical host-microbe interfaces for co-opting eukaryotic processes to create a niche for intracellular survival. Functionally characterizing such effectors is key to understanding these organisms' pathobiology. *Anaplasma phagocytophilum*, a member of the family *Anaplasmataceae*, is a tick-transmitted obligate intracellular bacterium and the etiologic agent of granulocytic anaplasmosis, an emerging, and debilitating human and veterinary infection in the United States, Europe, and Asia (Chen et al., 1994; Truchan et al., 2013; Bakken and Dumler, 2015). Human granulocytic anaplasmosis (HGA) manifests as an acute febrile disease that can be accompanied by leucopenia, thrombocytopenia, high levels of C-reactive protein and hepatic transaminases, and increased susceptibility to opportunistic infections. Risk for complications and fatality is greater for immunocompromised individuals, and when antibiotics are not promptly administered. *A. phagocytophilum* predominantly infects neutrophils (Bakken and Dumler, 2015), though endothelial cells have also been implicated as potentially being infected *in vivo* and have been shown to serve as reservoirs for passing the infection to myeloid cells under static and sheer flow conditions (Herron et al., 2005; Wang et al., 2015). A number of cell lines have proven useful for studying *A. phagocytophilum*-host cell interactions. HL-60 promyelocytic and RF/6A endothelial cells express known receptors that the bacterium uses for invasion and the latter is also large and flat, which facilitates easy visualization of the *A. phagocytophilum*-occupied vacuole (ApV; Goodman et al., 1996, 1999; Herron et al., 2000; Munderloh et al., 2004; Sukumaran et al., 2011; Niu et al., 2012; Ojogun et al., 2012; Seidman et al., 2015; Truchan et al., 2016a,b). HEK-293T cells are useful for studying the effects of transfected proteins on *A. phagocytophilum* because, in addition to supporting infection, these cells are much more amenable to transfection than HL-60 or RF/6A cells (Niu et al., 2012; Beyer et al., 2015; Truchan et al., 2016a,b).

Following invasion, *A. phagocytophilum* replicates, converts to the infectious form, and egresses from the host cell to initiate the next round of infection. The infection cycle takes 28–32 h to complete and the bacterium resides within the ApV for its entire intracellular life cycle (Troese and Carlyon, 2009). Only four bacterial proteins that localize to the ApV membrane (AVM) have been identified (Huang et al., 2010b,c; Sukumaran et al., 2011; Niu et al., 2012; Beyer et al., 2015). A domain that facilitates association with the AVM has yet to be delineated for these or any other *Anaplasmataceae* effector. We identified APH0032 as a protein that is expressed and pronouncedly localizes to the AVM late (24–28 h) in the infection cycle (Huang et al., 2010b). It is detectable on the AVM in neutrophils recovered from infected mice as well as infected myeloid, endothelial, HEK-293T, and tick embryonic cell lines (Huang et al., 2010b; Truchan et al., 2016b). Sera from HGA patients and animals experimentally infected with *A. phagocytophilum* recognize recombinant APH0032 (Storey et al., 1998; Huang et al., 2010b). Also, the bacterium transcribes *aph0032* during residence in tick salivary glands (Huang et al., 2010b). While these findings collectively imply the pathobiological importance of APH0032, its function is unknown.

APH0032 is a 619 amino acid protein that has a predicted molecular weight of 66.1 kDa. Amino acids 313 to 597 constitute a tandem repeat region composed of eight direct repeats that range in length from 33 to 35 amino acids and are preceded by a truncated segment that is homologous to the last 10 residues of each repeat (Huang et al., 2010b). APH0032 has been referred to as P130 due to its electrophoretic migration as a band having an apparent molecular weight of ~130 kDa along with several less abundant bands of ~66–200 kDa (Storey et al., 1998; Huang et al., 2010b). Its anomalous migration upon sodium dodecyl sulfate polyacrylamide gel electrophoresis (SDS-PAGE) is at least partially attributable to its acidic pI of 3.6. Other acidic proteins, including another *A. phagocytophilum* tandem repeat-containing protein (TRP), AmpA (*A. phagocytophilum* post-translationally modified protein A), and TRPs of the related *Anaplasmataceae* pathogen, *Ehrlichia chaffeensis*, bind SDS poorly and consequently migrate in polyacrylamide gels with apparent molecular weights that are aberrantly high (Storey et al., 1998; Huang et al., 2010b,c; Wakeel et al., 2010; Beyer et al., 2015). Of note, however, while multiple APH0032 isoforms are detected in Western-blotted lysates of *A. phagocytophilum* infected host cells, recombinant APH0032 expressed in *Escherichia coli* migrates as a single band (Storey et al., 1998; Huang et al., 2010b). This discrepancy suggests that the multiple isoforms might be due to APH0032 being post-translationally modified during *A. phagocytophilum* infection. Support for this rationale comes from our recent discovery that native AmpA, which also migrates as multiple isoforms upon SDS-PAGE, is SUMOylated during infection (Beyer et al., 2015).

SUMOylation is the covalent attachment of a member of the small ubiquitin-like modifier (SUMO) family of proteins to lysine residues in protein substrates. This pathway is an essential post-translational modification in eukaryotes and a major regulator of protein function, targeting thousands of proteins involved in diverse cellular processes by directly SUMOylating them or promoting protein-protein interactions that are mediated by SUMOylation. Cellular processes modulated by SUMOylation include antimicrobial pathways, RNA processing, chromatin remodeling, genome maintenance, transcriptional regulation, mitosis, meosis, differentiation, apoptosis, nucleocytoplasmic transport, and receptor trafficking (Wilkinson and Henley, 2010; Flotho and Melchior, 2013; Khan et al., 2016). Links between dysregulation of SUMOylation and cancer, inflammation, neurodegenerative disorders, and heart failure are established and, as such, SUMOylation is garnering attention as a potential therapeutic target (Flotho and Melchior, 2013; Khan et al., 2016). A consensus SUMOylation motif has been identified as ΨKxD/E, where Ψ is a bulky hydrophobic residue and x is any amino acid (Flotho and Melchior, 2013). However, not all lysines within such a motif are SUMOylated, and the modification can also occur outside this consensus (Wilkinson and Henley, 2010). The human genome encodes four distinct SUMO proteins. SUMO1-3 are ubiquitously expressed, while SUMO4 is specific to kidney, lymph nodes, and spleen (Citro and Chiocca, 2013). As these sites are irrelevant to the current study, SUMO4 will not be discussed further. SUMO2 and SUMO3 exhibit 97% sequence identity to each other and are collectively referred to as SUMO2/3. SUMO1 is distinct, exhibiting 47% identity to SUMO2/3 (Citro and Chiocca, 2013). SUMO2/3 moieties form polymers on substrates through the consensus site (Tatham et al., 2001; Pedrioli et al., 2006). SUMO1 was first shown to terminate poly-SUMO2/3 conjugates (Matic et al., 2008), but has since been demonstrated to also multimerize via non-consensus sites (Blomster et al., 2010; Galisson et al., 2011).

Microbial modulation of SUMOylation recently emerged as a conserved approach employed by viral and bacterial pathogens to inhibit the antimicrobial response and promote their survival (Hotson et al., 2003; Roden et al., 2004; Kim et al., 2008, 2013; Ribet et al., 2010; Wilson, 2012; Everett et al., 2013; Beyer et al., 2015; Wimmer and Schreiner, 2015). However, nearly all examples are of negative regulation of SUMOylation. Our recent discovery that AmpA is SUMOylated during *A. phagocytophilum* infection of mammalian cells marked the first examples of a bacterium co-opting SUMOylation to benefit its survival and of an *Anaplasmataceae* effector that is SUMOylated during infection (Beyer et al., 2015). Pharmacologic inhibition of SUMOylation drastically inhibits *A. phagocytophilum* intracellular growth (Beyer et al., 2015), suggesting that AmpA may not be the only effector of the bacterium that co-opts the post-translational process to benefit infection.

Herein, we identify the APH0032 domain that is essential for associating with eukaryotic membranes and confirm that it is presented on the AVM's cytosolic face, thus positioning the effector to interface with host cell processes. We demonstrate that native APH0032 is coprecipitated with SUMO capture beads during *A. phagocytophilum* infection of mammalian host cells, colocalizes with SUMO moieties on the AVM, and is important for *A. phagocytophilum* infection. This work ascribes functional relevance to APH0032 during infection by this obligate intracellular bacterium.

## Materials and methods

### Cell lines and *in vitro* cultivation of *A. phagocytophilum*

Uninfected and *A. phagocytophilum* (NCH-1 strain) infected HL-60, RF/6A, and HEK-293T cells were cultivated as described (Huang et al., 2012; Beyer et al., 2015; Truchan et al., 2016b). HeLa cells were cultivated as described (Beyer et al., 2015).

### Plasmid construction and transfection

APH0032 or fragments thereof were amplified using primers listed in Table 1 and Platinum Pfx DNA polymerase (Invitrogen, Carlsbad, CA). Amplicons were purified using a QIAquick PCR purification column (QIAGEN, Valencia, CA) and cloned into pENTR/TEV/D-Topo (Invitrogen) as described previously (Huang et al., 2010c) to yield pENTR entry plasmids containing the DNA fragment of interest. Plasmid inserts were verified by DNA sequencing, and recombination of the insert DNA downstream of and in frame with the gene encoding green fluorescent protein (GFP) in the pDest-53 plasmid (Invitrogen) was achieved using LR Clonase (Invitrogen) as described (Huang et al., 2010c). APH0032 K^*^R was generated by gene synthesis of APH0032 with the codons for all 6 lysine residues substituted with codons encoding arginine and flanked with EcoRI and SalI restriction sites (Biomatik, Ontario, Canada). APH0032 K^*^R DNA was restriction cloned into the peGFP-NZ plasmid (Clonetech, Palo Alto, CA) via the EcoRI/SalI sites and sequenced to confirm insert integrity. All plasmids expressed APH0032 proteins N-terminally fused to GFP.

**Table 1:** **Oligonucleotides used in this study**.

**Designation**	**Sequence (5′ to 3′)^a^**	**Targeted nucleotides^b^**
*aph0032*-4F-ENTR	**CACC**TTTGAACACAATATTCCTGATACATACACAGG	4–35 (+)
*aph0032*-1860R	TCACAACGCGAGCACGTCATC	1840–1860 (−)
*aph0032*-1F-ENTR	**CACC**ATGTTTGAACACAATATTCCTGATAC	1–26 (+)
*aph0032*-936R	CTATAAAGGCAATGTACCTAGTTCCTGAACATTGTC	904–936 (−)
*aph0032*-904F-ENTR	**CACC**GACAATGTTCAGGAACTAGGTACATTGCC	904–932 (+)
*aph0032*-426R	CTACGGTTGAGGAGCTACTTCCTCG	405–426 (−)
*aph0032*-525R	CTAAGTATCGCTACTATTACACTTGCTGTCTTCAGC	493–525 (−)
*aph0032*-403F-ENTR	**CACC**GCCGAGGAAGTAGCTCCTCAACC	403–425 (+)
*aph0032*-487F-ENTR	**CACC**GACACTGCTGAAGACAGCAAGTGTAATAGTAG	487–518 (+)
*Ap 16S*-527F	TGTAGGCGGTTCGGTAAGTTAAAG	527–550 (+)
*Ap 16S*-753R	GCACTCATCGTTTACAGCGTG	733–753 (−)
β*-actin-2133F*	AGAGGGAAATCGTGCGTGAC	2133–2152 (+)
β*-actin-2270R*	CAATAGTGATGACCTGGCCGT	2250–2270 (−)

a Nucleotides in bold text correspond to a Gateway entry vector-compatible sequence; underlined nucleotides correspond to an added stop codon.

b (+), sense strand; (−) antisense strand.

### Post-digitonin permeabilization retention assay

HeLa cells seeded on 12-mm glass coverslips were transfected with 0.4 μg of endotoxin-free purified plasmid DNA encoding GFP-tagged APH0032 or portions thereof using Lipofectamine 2000 (Invitrogen) according to the manufacturer's directions. At 24 h, the cells were washed three times with phosphate buffered saline (PBS) and then once with digitonin buffer (125 mM NaC_2_H_3_O_2_, 2.5 mM Mg[CH_3_COO]_2_, 25 mM HEPES-KOH, 1 mg ml^−1^ glucose, 1 mM dithiothreitol, pH 7.3). Cells were subsequently incubated with digitonin buffer with or without 30 μg ml^−1^ digitonin (Sigma, St. Louis, MO) for 5 min to permeabilize the host cell plasma membrane, but not organelle membranes (Checroun et al., 2006), followed by two washes with digitonin buffer to remove any free GFP fusion protein. Cells were fixed in 4% paraformaldehyde (PFA) in PBS for 1 h at room temperature and permeabilized in ice-cold methanol for 30 s. Coverslips were mounted with ProLong Gold Antifade (Invitrogen) containing 4′, 6′-diamidino-2-phenylindole (DAPI) and slides were analyzed via spinning-disc confocal microscopy (Huang et al., 2010a). Images were processed using the Slidebook software package (Intelligent Imaging Innovations, Denver, CO). One hundred cells were examined per condition. The percentage of each GFP fusion protein that was retained by HeLa cells following digitonin permeabilization was calculated by dividing the number of GFP-positive, digitonin-treated cells by the number of GFP-positive, vehicle control-treated cells and multiplying the quotient by 100.

### Assay for determining if APH0032 immunoaccessible domains are on the AVM cytosolic face

RF/6A cells were synchronously infected as described previously (Huang et al., 2012). To determine if immunoaccessible domains of APH0032 were present on the cytosolic face of the AVM, the cells were permeabilized at 24 h post-infection with digitonin or saponin according to a protocol established by Checroun, Celli, and colleagues for assessing pathogen-occupied vacuole integrity (Checroun et al., 2006). Digitonin permeabilizes the plasma membrane but not organelle membranes to enable antibody delivery to the cytoplasm (Checroun et al., 2006), whereas saponin permeabilizes plasma and organelle membranes to allow for antibody delivery inside organelles (Checroun et al., 2006; Hasegawa et al., 2009). Cells were washed twice with digitonin buffer followed by continued incubation in the presence of 30 μg ml^−1^ digitonin or 0.1% saponin. The cells were immediately washed with digitonin buffer and incubated for 30 min at 37°C with rabbit polyclonal antiserum targeting APH0032 (Huang et al., 2010a,c) at a 1:500 dilution in PBS containing 1% bovine serum albumin (BSA). To confirm that digitonin permeabilized the plasma membrane without compromising the integrity of the AVM, digitonin-treated cells were incubated with antiserum specific for the *A. phagocytophilum* major surface protein, P44, which does not localize to the AVM (Huang et al., 2010c). Bound primary antibodies were detected by Alexa Fluor 594-conjugated goat anti-rabbit IgG (Invitrogen) and spinning disc confocal microscopy.

### *In silico* analyses

The APH0032 sequence (APH0032; APH_RS00145) was analyzed for predicted SUMOylation sites using GPS-SUMO (http://sumosp.biocuckoo.org/; Zhao et al., 2014) and PCI-Based Sumo Site Prediction Server (PCI-SUMO; http://bioinf.sce.carleton.ca/SUMO/start.php).

### Analysis of APH0032 colocalization with SUMO-1 and SUMO-2/3 in *A. phagocytophilum*-infected cells

*A. phagocytophilum* infected RF/6A cells grown on coverslips were fixed and processed for laser scanning confocal microscopy (LSCM). To generate murine polyclonal APH0032 antiserum, C3H/HeJ mice were immunized against glutathione-*S*-transferase-tagged APH0032 as previously described (Troese et al., 2011) in accordance with the recommendations of and following a protocol approved by the University of California Davis Institutional Animal Care and Use Committee. Cells were probed with APH0032 antiserum and rabbit anti-SUMO1 (AbCam, Cambridge, MA) or rabbit anti-SUMO2/3 (AbCam) antibody as previously described (Beyer et al., 2015). Alexa Fluor 594-conjugated goat anti-rabbit IgG (Invitrogen) and Alexa Fluor 488 conjugated goat anti-mouse IgG (Invitrogen) were used as secondary antibodies prior to mounting as described above. Cells were imaged with a Zeiss LSM 700 laser-scanning confocal microscope.

### *In vivo* SUMOylation assay

*A. phagocytophilum*-infected HL-60 cells were homogenized in lysis buffer (50 mM Tris-HCl, pH7.5, 150 mM NaCl, and 200 mM iodoacetamide [Sigma]) with protease inhibitor cocktail (Roche Diagnostics GmBH, Mannheim, Germany). One hundred micrograms of each lysate was subjected to SUMO-Qapture-T pulldowns (Enzo Life Sciences, Farmingdale, NY, USA) following manufacturer's directions. Eluates were resolved by SDS-PAGE and transferred to polyvinyl difluoride membrane (PVDF; BioRad). Blots were blocked in Tris-buffered saline (TBS) containing 5% non-fat milk and probed as described (Truchan et al., 2016b). Blots were screened with rabbit anti-APH0032 (Huang et al., 2010b), mouse anti-APH1235 (Troese et al., 2011), or rabbit anti-Ubc9 (Santa Cruz Biotechology, Dallas, TX). Secondary antibodies were horseradish peroxidase-linked goat anti-rabbit IgG or anti-mouse IgG (Cell Signaling technology, Beverly, MA).

### Assessment of APH0032 ectopic expression on *A. phagocytophilum* infection

HEK-293T were synchronously infected with host cell-free *A. phagocytophilum* organisms that had been naturally released from infected RF/6A cells as previously described (Truchan et al., 2016b). At 17 h post-infection, the cells were transfected to express GFP, GFP-APH0032, or GFP-APH0032 K^*^R. At 11 h post-transfection (28 h post-infection), cells were fixed and analyzed by immunofluorescent microscopy to determine the percentage of infected GFP-positive cells. For quantitative PCR (QPCR) analysis, GFP expressing cells were isolated using a BSC Aria-BD FACS Aria II High-Speed Cell Sorter. Total DNA was isolated from each sorted sample using the DNeasy Blood and Tissue kit (QIAGEN). QPCR was performed on triplicate samples consisting of 100 ng genomic DNA, SsoFast Evagreen Supermix (Bio-Rad), and primers targeting genes encoding *A. phagocytophilum* 16S rDNA and human β-actin (Table 1). QPCR assays were performed using the CFX96 real-time PCR detection system (Bio-Rad). Relative infection load levels were normalized to the DNA levels of the host cell β-actin gene using the 2−ΔΔCT (Livak) method (Livak and Schmittgen, 2001) and the Prism 5.0 software package (Graphpad, San Diego, CA).

### Statistical analyses

Statistical analyses were performed using the Prism 5.0 software package (Graphpad, San Diego, CA). If One-Way Analysis of Variance (ANOVA) indicated a group difference (*P* < 0.05), then Dunnet's or Tukey's *post-hoc* test was used to test for a significant difference among groups.

## Results

### Delineation of the APH0032 region that facilitates association with mammalian host cell membranes

**Figure 1:**
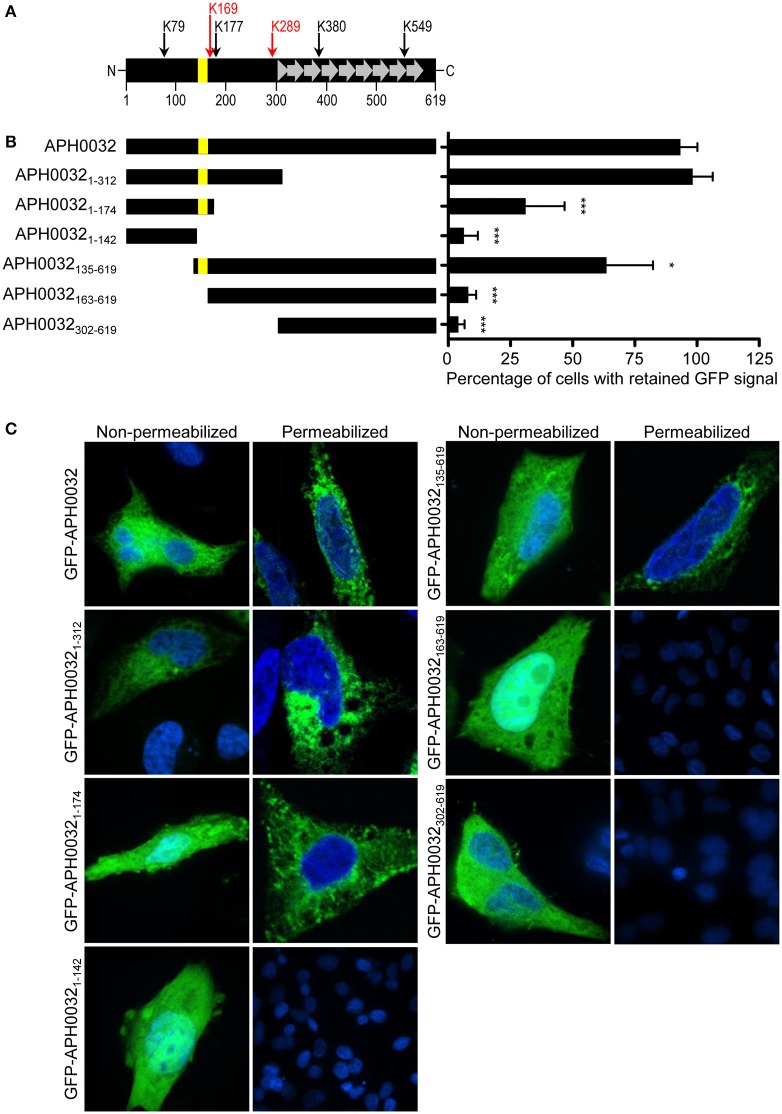
**Identification of the APH0032 region that facilitates association with mammalian host cell membranes. (A)** Schematic representation of the APH0032 sequence. The amino (N)-terminal region (amino acids 1–312) precedes the carboxy (C)-terminal region (amino acids 313–619), the latter of which carries a series of direct repeats arranged in tandem (amino acids 313–597). There are eight imperfect direct repeats (designated by gray arrows) that range in size from 33 to 35 amino acids. The eight direct repeats are preceded by a truncated segment that is homologous to the last 10 amino acids of each repeat (gray triangle). The yellow bar represents the predicted transmembrane domain encoded by amino acids 144–162. The numerical scale denotes the protein sequence at 100-amino acid intervals. Arrows above the diagram indicate the six lysine (K) residues present within APH0032 and their amino acid positions. Red arrows indicate lysine residues that are predicted to be SUMOylated. **(B)** Schematic representations of GFP-APH0032 truncation constructs and graphic display of the percentage of cells expressing GFP-APH0032 proteins that retained GFP signal following digitonin permeabilization. The construct schematics are scaled proportionally to the schematic in panel A, and the predicted transmembrane domain, if present, is indicated per construct (yellow bar). Duplicate sets of HeLa cells were transfected to express GFP-tagged APH0032 or portions thereof, incubated with digitonin or vehicle control, washed, fixed, and examined by confocal microscopy. The percentages of digitonin-permeabilized cells that retained GFP-APH0032 proteins relative to control cells were calculated. One hundred cells were examined per condition. Statistical significance among groups was determined using One-Way ANOVA followed by Dunnet's *post-hoc* test. Statistically significant (*P < 0.05; ***P < 0.0001) values are indicated. **(C)** Representative confocal micrographs showing retention, or lack thereof, of GFP-APH0032 proteins in non-permeabilized and digitonin permeabilized HeLa cells. Cell nuclei are stained with DAPI. Results presented are the mean ± *SD* of three independent experiments.

How APH0032 associates with the AVM is unknown. Because it is predicted to carry a hydrophobic alpha-helical transmembrane domain (TMD) that spans residues 144–162 (Huang et al., 2010a), an assay was developed to determine if this or any other APH0032 region facilitates association with mammalian cell membranes. HeLa cells were transfected to express GFP-tagged APH0032 or portions thereof (Figure 1A). At 24 h, duplicate sets of transfected cells were incubated with digitonin or vehicle control, washed, and examined by LSCM. Given that digitonin permeabilizes plasma membranes but not organelle membranes (Checroun et al., 2006), GFP-fusion proteins not associated with organelle membranes would be flushed out of digitonin-permeabilized but not control-treated cells during the washing steps. The percentage of each GFP-APH0032 protein that was retained by HeLa cells following digitonin permeabilization was calculated by dividing the number of GFP-positive, digitonin-treated cells by the number of GFP-positive, vehicle control-treated cells and multiplying the quotient by 100. GFP-tagged APH0032, APH0032_1–312_, and APH0032_135–619_, each of which carried the predicted transmembrane domain, were retained by 93.3 ± 6.7, 98.3 ± 8.0, and 63.7 ± 18.6%, respectively, of HeLa cells following permeabilization (Figures 1B,C). GFP-APH0032_1–174_, which carries the putative transmembrane domain plus an additional 12 C-terminal residues, exhibited only 31.2 ± 15.6 retention (Figures 1B,C). GFP-APH0032 fusions lacking the predicted transmembrane domain exhibited little to no retention (Figures 1B,C). Thus, the APH0032 region that promotes optimal association with eukaryotic membranes includes the predicted transmembrane domain of residues 144 to 162.

### APH0032 is presented on the cytosolic face of the AVM

**Figure 2:**
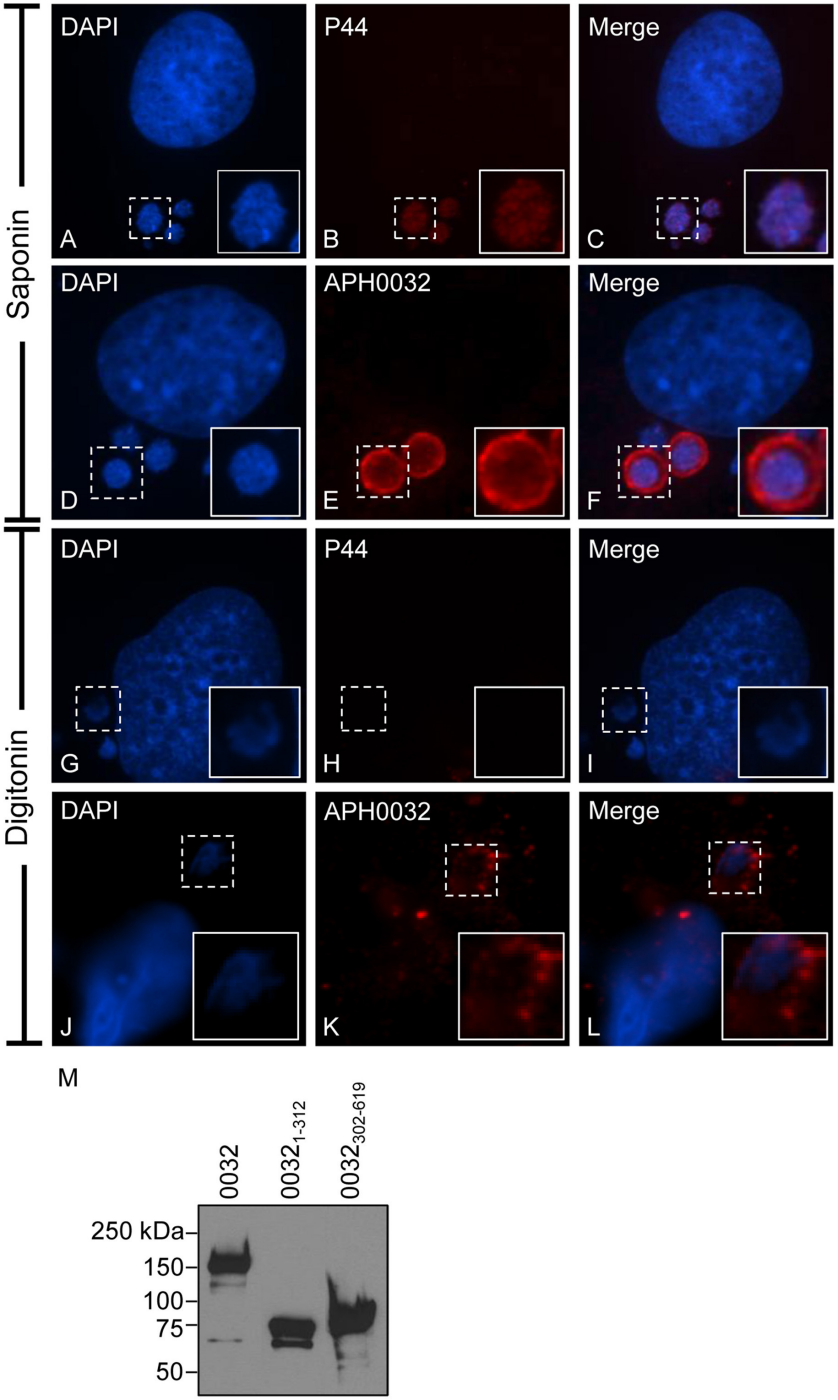
**APH0032 is exposed on the cytosolic face of the AVM**. *A. phagocytophilum* infected RF/6A cells were permeabilized with either saponin **(A–F)**, which permeabilizes both the plasma and organelle membranes to allow for antibody delivery inside organelles, or digitonin **(G–L)**, which permeabilizes the plasma membrane but not organelle membranes. The cells were screened with APH0032 antiserum **(D–F, J–L)**, fixed, and viewed by indirect immunofluorescence confocal microscopy. To verify that digitonin permeabilized the plasma membrane without compromising the integrity of the AVM, digitonin-treated cells were incubated with a monoclonal antibody specific for P44 **(A–C, G–I)**, which is found on the bacterium's surface but not on the AVM. Host cell and bacterial nuclei are stained with DAPI **(A,D,G,J)**. Bound primary antibodies were detected by Alexa Fluor 594-conjugated goat anti-rabbit IgG **(B,E,H,K)** and confocal microscopy. Merged images are presented in panels **(C,F,I,L)**. Corner inset images are enlarged views of the regions denoted by hatched line boxes. **(M)** APH0032 antiserum recognizes both the non-repeat and tandem repeat portions of APH0032. Whole cell lysates of HeLa cells transfected to express GFP-tagged APH0032 or portions thereof were Western blotted and screened with APH0032 antiserum. Results are representative of at least two separate experiments with similar results.

For APH0032 to serve as an interface with host cell factors, it would need to be exposed on the cytosolic face of the AVM. To assay for this phenomenon, we incubated *A. phagocytophilum* infected RF/6A cells with saponin or digitonin prior to fixation. Saponin permeabilizes the plasma and cholesterol-rich intracellular membranes to enable antibody delivery within organelles, including pathogen-occupied vacuoles (Checroun et al., 2006; Hasegawa et al., 2009). Digitonin selectively permeabilizes the plasma membrane, which allows for antibody delivery to the cytoplasm and to the cytosolic faces of organelles but not within organelles (Checroun et al., 2006). Control antiserum against the *A. phagocytophilum* outer membrane protein, P44, detected bacteria within vacuoles of saponin- but not digitonin-treated cells (Figures 2A–C,G–I), verifying that intravacuolar bacteria are inaccessible to antibodies delivered into the cytosol. APH0032 antiserum partially stained intravacuolar bacteria and pronouncedly stained the AVM in saponin-permeabilized cells (Figures 2D–F). Consistent with our previous report of the temporal nature by which APH0032 is differentially expressed throughout *A. phagocytophilum* intracellular development (Huang et al., 2010a), not all ApVs were APH0032-positive. APH0032 antibody recognized its protein target on the AVM but did not detect intravacuolar *A. phagocytophilum* organisms in digitonin-treated cells (Figures 2J–L), indicating that APH0032 or at least a portion thereof is exposed on the AVM's cytosolic face.

To determine which portion or portions of APH0032 the antiserum recognized, Western blot analyses were performed on whole cell lysates of transfected HeLa cells expressing GFP-tagged full-length APH0032 or the non-repeat region (APH0032_1–312_) or tandem repeat region (APH0032_302–619_) thereof. Consistent with previous reports for endogenous APH0032 and other TRPs (Storey et al., 1998; Huang et al., 2010b,c; Wakeel et al., 2010; Beyer et al., 2015), GFP-APH0032 proteins yielded anomalous electrophoretic migration patterns (Figure 2M). APH0032 antiserum recognized all three recombinant proteins, indicating that the antiserum does not discriminate between the non-repeat and tandem repeat regions.

### *In silico* analyses identify potential APH0032 sumoylation sites

SUMOylated proteins are detected on or in very close proximity to the AVM (Beyer et al., 2015). Because APH0032 is presented on the AVM's cytosolic face where it is poised to interface with host cell factors, it contains six lysine residues, and lysines are frequent targets for the covalent attachment of SUMO moieties (Wilkinson and Henley, 2010), its sequence was examined for the presence of potential SUMOylation motifs using prediction algorithms. Two lysine residues were predicted to be SUMOylated based on similarity to the ΨKxD/E consensus motif and to other residues known to flank such SUMOylation sites. K289, which lies just upstream of the TMD (Figure 1A) was predicted by both GPS-SUMO (http://sumosp.biocuckoo.org/; Zhao et al., 2014) and PCI-SUMO (http://bioinf.sce.carleton.ca/SUMO/start.php) to be a SUMOylation target, whereas K169, which lies just downstream of the eukaryotic membrane localization region (Figure 1A), was predicted by only PCI-SUMO to be SUMOylated. The remaining APH0032 lysines (K79, K177, K380, and K546) were not predicted to be sites of SUMOylation. Given that a function had yet to be ascribed to APH0032, it was next investigated whether it co-opts SUMOylation.

### A SUMO affinity matrix precipitates native APH0032 from *A. phagocytophilum* infected host cells

**Figure 3:**
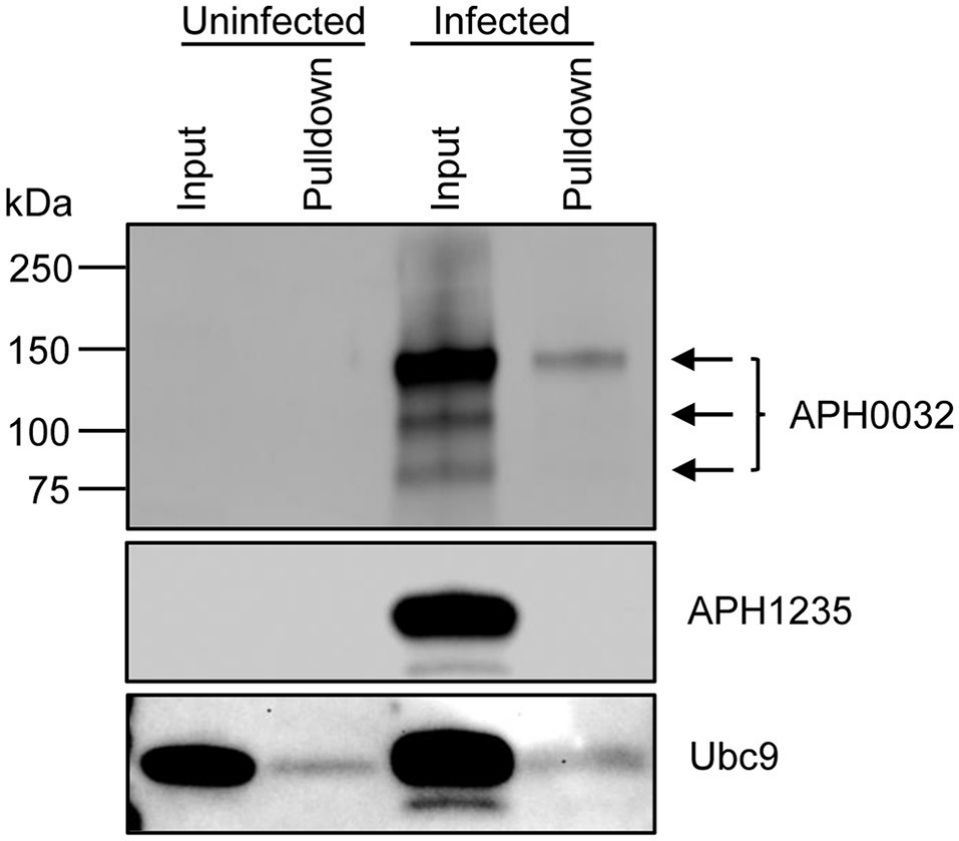
**Native APH0032 expressed by *A. phagocytophilum* is SUMOylated during infection of mammalian host cells**. Uninfected and *A. phagocytophilum* infected HL-60 cells were lysed and incubated with SIM beads. Western-blotted eluted proteins (pulldown) and input lysates were screened with antibodies against APH0032, the negative control APH1235, and positive control Ubc9. Arrows denote the multiple isoforms of APH0032. Data presented are representative of three experiments with similar results.

Lysates of uninfected and *A. phagocytophilum* infected HL-60 cells were incubated with beads that were coupled with SUMO interaction motifs (SIMs) to precipitate SUMOylated proteins. Western blots of the resulting eluted proteins and input lysates were screened with antibodies against APH0032, Ubc9, or APH1235, the latter two of which were positive and negative controls, respectively. Ubc9 is an E2 ligase of SUMOylation that is itself a substrate for SUMO modification (Wilkinson and Henley, 2010). *A. phagocytophilum* expresses APH1235 during the same late infection cycle time period as APH0032, but is not an effector, does not localize to the AVM (Troese et al., 2011; Mastronunzio et al., 2012), and therefore is not expected to be accessible to host cell SUMO ligases. Ubc9 was detected in all input and pulldown samples, while APH1235 was detected only in the input from infected cells (Figure 3). Similar to previous reports (Storey et al., 1998; Huang et al., 2010b), multiple APH0032 bands of varying apparent molecular weights, the most predominant of which was ~130 kDa, were detected in the infected sample input. APH0032 was recovered by SIM affinity beads as a minor portion of the total APH0032 protein content and migrated with an apparent molecular weight of 130 kDa. Thus, endogenous APH0032 expressed by *A. phagocytophilum* during infection of mammalian cells is either SUMOylated or interacts with a SUMOylated host protein.

### APH0032 on the AVM predominantly colocalizes with SUMO1 moieties

**Figure 4:**
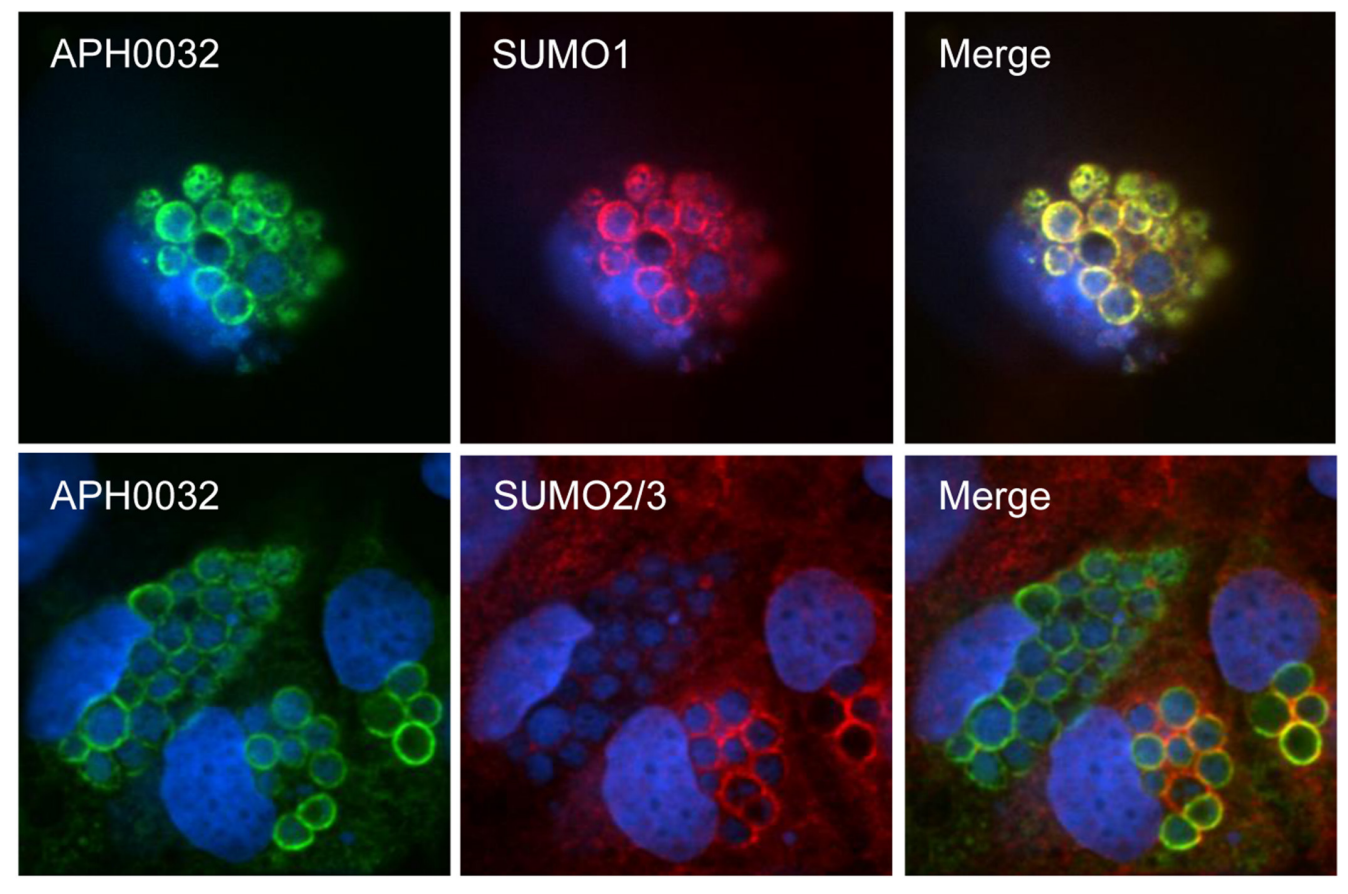
**APH0032 on the AVM preferentially colocalizes with SUMO1 moieties**. RF/6A cells were synchronously infected with *A. phagocytophilum*. At 28 h, the cells were fixed, screened with antibodies against APH0032 and SUMO2/3 or SUMO1, stained with DAPI, and imaged using LSCM. Merged images are shown in the third column. Results are representative of three experiments with similar results.

SUMO2/3 and SUMO1 moieties are detected on or in close proximity to the cytosolic face of the AVM (Beyer et al., 2015). Given that APH0032 co-opts SUMOylation, infected RF/6A cells were examined by LSCM for colocalization of SUMO2/3 or SUMO1 moieties with APH0032 on the AVM. Whereas SUMO2/3 signal colocalized with AVM-associated APH0032 signal at distinct points on some ApVs, SUMO1 and APH0032 signals colocalized perfectly on all APH0032-positive ApVs (Figure 4). Thus, APH0032 preferentially localizes with SUMO1 moieties on the AVM.

### APH0032 promotes *A. phagocytophilum* infection

**Figure 5:**
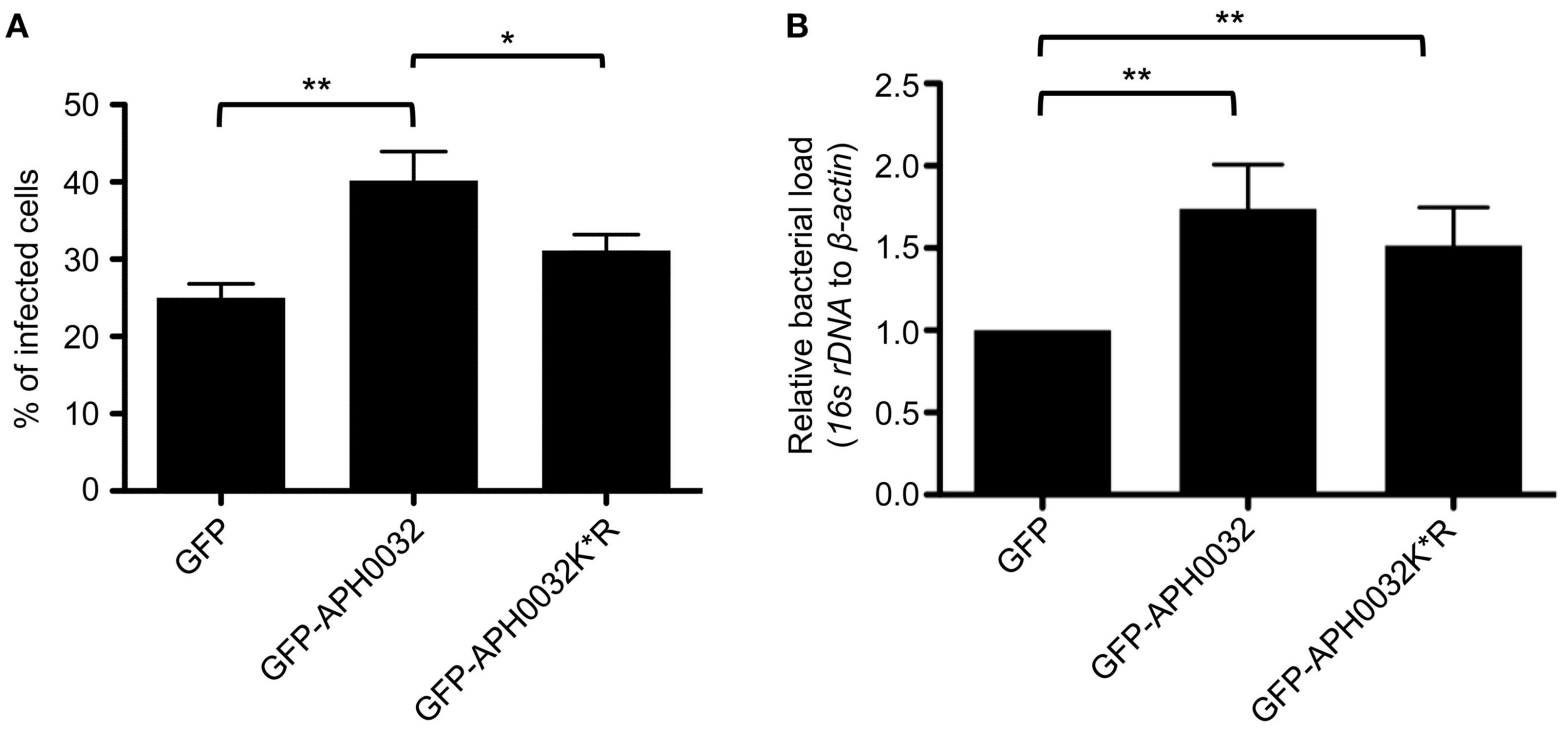
**Ectopically expressed APH0032 results in a boost in bacterial infection**. HEK-293T cells were synchronously infected with *A. phagocytophilum*. At 17 h post-infection, the cells were transfected to express GFP, GFP-APH0032, or GFP-APH0032 K*R. At 28 h post-infection, cells were either analyzed by immunofluorescence microscopy **(A)** or sorted for GFP expression using a cell sorter followed by DNA isolation from each respective sorted fraction **(B)**. QPCR was performed and the bacterial DNA load was determined by normalizing the relative amount of *A. phagocytophilum 16S* to host β*-actin* DNA using the Livak method in populations of GFP-positive cells. Data in panel B are presented as the fold change relative to GFP control. Statistical significance among groups was determined using One-Way ANOVA followed by Tukey's *post-hoc* test. Statistically significant (*P < 0.05; **P < 0.005) values are indicated. Data represents the mean ± *SD* of two independent experiments performed in duplicate and triplicate.

Because APH0032 co-opts SUMOylation, a process that is critical for optimal *A. phagocytophilum* replication (Beyer et al., 2015), and localizes to bacterial inclusions, it was next examined if APH0032 affects *A. phagocytophilum* infection. As genetic manipulation of *A. phagocytophilum* at a specific locus is not yet possible, this question could not be answered using knock out-complementation. Rather, the effect of ectopically expressed APH0032 on the bacterial load was determined. HEK-293T cells that had been synchronously infected with *A. phagocytophilum* were transfected to express GFP-tagged APH0032 or GFP alone. To confirm whether any observed effect is dependent on APH0032 lysine residues, the experiment was also performed on cells expressing GFP-APH0032 K^*^R in which all six lysines were substituted with arginine, an amino acid that is structurally similar to lysine but cannot be SUMOylated. At 11 h post-transfection, which corresponded to the 28 h infection cycle time point when the bacterium pronouncedly expresses APH0032 (Huang et al., 2010b), GFP-positive cells were either examined by immunofluorescence microscopy to determine the percentage of infected cells or were isolated using fluorescence activated cell sorting and subjected to QPCR to quantify the bacterial DNA load. Relative to cells expressing GFP, both the percentage of infected cells and *A. phagocytophilum* DNA load were increased 1.6-fold in cells expressing GFP-APH0032 (Figure 5). Similar results were observed for cells expressing GFP-APH0032 K^*^R, though the increases were not as substantial as those for GFP-APH0032 expressing cells. Thus, APH0032 enhances *A. phagocytophilum* infection and intracellular replication and its ability to optimally do so involves one or more of its lysines.

## Discussion

For a bacterial pathogen-occupied vacuole membrane protein to co-opt host cellular processes, it needs to associate with the membrane in a manner that presents at least a portion of it on the vacuole's cytosolic face. This would involve either a hydrophobic TMD that traverses the membrane or a domain or post-translational modification that facilitates peripheral association with one or more of membrane lipids or proteins. In this study, we delineated the APH0032 region that is essential for optimally associating with eukaryotic membranes as including residues 144–162, which are predicted to form a TMD (Huang et al., 2010b). This is the first such region identified for any *Anaplasmataceae* effector. APH0032 is predicted to traverse the AVM only once, which would place its portion that is either N- or C-terminally located relative to the TMD on the ApV's cytosolic face as a platform for interacting with host cellular processes. APH0032 antiserum immunolabels the cytosolic faces of non-permeabilized ApVs and antiserum more effectively detects its target on saponin-permeabilized ApVs. The latter result could be due to APH0032 antibodies recognizing epitopes present on both the cytosolic and lumenal faces of the AVM. If APH0032 was a peripheral membrane protein, then immunolabeling would be expected to be consistent between saponin- and digitonin-permeabilized ApVs. Tandem repeat regions of *E. chaffeensis* TRPs are responsible for mediating protein-protein interactions (Luo et al., 2011; Dunphy et al., 2014; Lina et al., 2016b). Based on this precedent our findings, we favor a model in which APH0032 is positioned in the AVM by virtue of its TMD such that its hydrophilic C-terminal tandem repeat-containing portion is exposed to the cytosol.

*A. phagocytophilum* TRPs APH0032 and AmpA and *Ehrlichia* spp. TRPs lack sequence homology, but share a bipartite architecture that consists of N-terminal non-repeat and C-terminal acidic tandem repeat-containing regions (Huang et al., 2010b,c; Luo et al., 2011; McBride et al., 2011; Wakeel et al., 2011; Luo and McBride, 2012; Lina et al., 2016b). These effectors' importance to *A. phagocytophilum*- and *Ehrlichia* spp.-host cell interactions are just beginning to be appreciated. While several bacteria have been shown to modulate SUMOylation during infection (Hotson et al., 2003; Roden et al., 2004; Kim et al., 2008, 2013; Ribet et al., 2010; Wilson, 2012; Everett et al., 2013; Beyer et al., 2015; Wimmer and Schreiner, 2015), AmpA was the first obligate intracellular bacterial effector proven to be SUMOylated during infection (Beyer et al., 2015). APH0032 also co-opts SUMOylation, as evidenced by the fact that it can be precipitated from a lysate of *A. phagocytophilum* infected cells by beads coated with SUMO interaction motifs. Whether APH0032 is directly SUMOylated or whether it is co-precipitated by virtue of interacting with a SUMOylated protein remains to be discerned. The susceptibility of *A. phagocytophilum* to the SUMOylation inhibitor, anacardic acid (Beyer et al., 2015), demonstrates that hijacking the post-translational process, likely by APH0032, AmpA, and possibly unidentified effectors is critical for the bacterium's intracellular survival. The specific advantage that SUMOylation provides *A. phagocytophilum* is unknown. Notably, individual ehrlichial TRPs are capable of interacting with an array of host proteins that localize to the pathogen-occupied vacuole and regulate many cellular processes including signal transduction, vesicular trafficking, transcriptional regulation, translation, proteome mediated-degradation, metabolism, and cytoskeletal organization (Luo et al., 2011; Luo and McBride, 2012; Dunphy et al., 2014; Lina et al., 2016a,b). *E. chaffeensis* growth is also suppressed by anacardic acid, and recombinant forms of ehrlichial TRPs or peptides thereof are SUMOylated *in vitro* and/or when ectopically expressed (Dunphy et al., 2014; Lina et al., 2016b; Zhu et al., 2016). Given that SUMOylation promotes protein-protein interactions, it is likely that co-opting SUMOylation is essential for APH0032 and AmpA function and doing so enables these and ehrlichial TRPs to modulate numerous cellular processes that are important for survival.

Like AmpA (Beyer et al., 2015), the small amount of native APH0032 recovered by SUMO affinity beads indicates that only a minor proportion of the protein co-opts SUMOylation at any given time. This finding evokes the “SUMO enigma,” which is a phenomenon that occurs in eukaryotic cells by which only a small portion of an available substrate need to be SUMOylated to achieve maximal effect (Hay, 2005). SUMO1-modified proteins decorate the AVM only between 24 and 28 h, a time period that conspicuously corresponds to when APH0032 is expressed (Huang et al., 2010b; Beyer et al., 2015). Moreover, APH0032 colocalizes perfectly with SUMO1 moieties on the AVM. The few instances of APH0032-SUMO2/3 colocalization that were inconsistently observed for some ApVs may be simply due to the fact that SUMO2/3-modified proteins pronouncedly label the AVM throughout infection (Beyer et al., 2015). SUMO1 moieties can either terminate poly-SUMO2/3 chains or can multimerize at non-consensus SUMOylation sites (Matic et al., 2008; Blomster et al., 2010; Galisson et al., 2011). Because APH0032 specifically colocalizes with SUMO1 but not SUMO2/3 moieties, it is reasonable to conclude that APH0032 may either be SUMO1-modified or associate with one or more SUMO1-modified proteins on the AVM. Overexpression of GFP-APH0032 in infected cells boosted the *A. phagocytophilum* load both in terms of the percentage of infected cells and bacterial DNA load. While it cannot be absolutely ruled out that this phenotype is due to ectopically expressed APH0032 having an off target effect on host cell function, this result most likely indicates that the native effector ultimately benefits bacterial intracellular replication. The increase in the percentage of infected cells at 28 h, a time point that normally corresponds to a single round of infection, is notable because it suggests that the *A. phagocytophilum* infection cycle may have been accelerated in cells ectopically expressing APH0032. This phenotype is similar to that observed for infected cells ectopically expressing GFP-tagged Ats-1 (*Anaplasma* translocated substrate 1), an *A. phagocytophilum* effector that enhances growth by inducing formation of autophagosomes that serve as an amino acid source for the bacterium (Niu et al., 2012). GFP-APH0032 K^*^R was less effective at augmenting infection. Given that the arginine for lysine substitutions likely did not affect APH0032 protein structure, this result indicates that lysines may be sites of an important post-translational modification, such as SUMOylation, or may contribute to optimal interaction with a host cell ligand.

In summary, we determined how an APH0032 associates with the ApV and demonstrated that it co-opts SUMOylation. Regardless of whether it does so by becoming directly SUMOylated like AmpA or by interacting with a host cell SUMOylated protein, our work reinforces the importance of SUMOylation to *A. phagocytophilum* infection. It will be important to identify the APH0032 host cell interacting partner(s), define how the interaction benefits infection, and resolve whether and, if so, how SUMOylation influences APH0032 function. This study adds to a growing body of literature indicating the importance of microbial co-option of SUMOylation and how critical this post-translational modification is to intracellular infection by *Anaplasmataceae* pathogens.

## Author contributions

JAC, ATO, BH, DCB designed experiments. ATO, BH, ARB, LJM, HKT, NJW, NLG performed the experiments. ATO, BH, ARB, HKT analyzed the data. JAC, ATO, HKT wrote the paper.

## Funding

This study was supported by funding from National Institutes of Health Grants R21 AI105364, R21 AI122014, R01 AI072683, National Center for Advancing Translational Sciences Grant UL1TR000058, and the Center for Clinical and Translational Research Endowment Fund of VCU (to JAC). LJM was supported by the VCU Summer Student Program in Microbiology, Infectious Diseases, and Public Health Epidemiology. LSCM was performed at the VCU Microscopy Facility, which is supported in part with funding from NIH-NINDS Center core grant 5P30NS047463 and NIH-NCI Cancer Center Support Grant (P30 CA016059). Cell sorting was performed at VCU Massey Cancer Center Flow Cytometry Shared Resources, which is supported in part with funding from NIH-NCI Cancer Center Support Grant (P30 CA016059).

### Conflict of interest statement

The authors declare that the research was conducted in the absence of any commercial or financial relationships that could be construed as a potential conflict of interest.
